# Some Aspects of Military Ophthalmology

**Published:** 1919-01

**Authors:** 


### OPHTHALMOLOGY AND OTO-LARYNGOLOGY

***Some Aspects of Military Ophthalmology.*** By Colonel S. Hanford McKee, C. A. M. C. *Bulletin of the Canadian Medical Corps*, September, 1918.

The author speaks of the difference between military and civilian ophthalmology, and insists upon the desirability of having, in every military hospital, a special ophthalmologic section for the examination and classification of recruits.

A series of observations is then given, which may be summarized as follows:

In some cases of myopia and astigmatism the patient's vision was normal before proceeding to France, and the defect was probably due to high explosives. Epidemic conjunctivitis and trachoma have almost disappeared as a military disease. Cases of gonorrheal ophthalmia have also been very few in this campaign. Night blindness, on the other hand, has taken a prominent part; in a number of cases it is associated with retinitis pigmentosa. Out of 105 cases of iritis, Wassermann's reaction was positive in 33 and negative in 72; in 4 cases of retinitis pigmentosa the reaction was negative; in 11 cases of optic neuritis, 5 were positive, 6 negative; in 7 cases of interstitial keratitis, the reaction was positive in all; in 30 cases of retino-choroiditis 15 were positive and 15 negative. Out of 3000 ophthalmic cases observed, only 2 were cases of sympathetic ophthalmia. A large number and variety of fundus lesions have been met with, the commonest being a condition of traumatic retino-choroiditis identical with retino-choroiditis of secondary syphilis. No attempt should be made to correct strabismus. The West operation is recommended for dacryocystitis.

The author further records the work of various Canadian Hospitals.

